# Peak Velocity as an Alternative Method for Training Prescription in Mice

**DOI:** 10.3389/fphys.2018.00042

**Published:** 2018-02-06

**Authors:** Caroline de Carvalho Picoli, Paulo Vitor da Silva Romero, Gustavo R. Gilio, Débora A. Guariglia, Laize P. Tófolo, Solange M. F. de Moraes, Fabiana A. Machado, Sidney B. Peres

**Affiliations:** ^1^Department of Physical Education, State University of Maringá, Paraná, Brazil; ^2^Department of Physiological Sciences, State University of Maringá, Paraná, Brazil

**Keywords:** treadmill, incremental test, running, training programs, exercise test

## Abstract

**Purpose:** To compare the efficiency of an aerobic physical training program prescribed according to either velocity associated with maximum oxygen uptake (vVO_2max_) or peak running speed obtained during an incremental treadmill test (V_peak_K_) in mice.

**Methods:** Twenty male Swiss mice, 60 days old, were randomly divided into two groups with 10 animals each: 1. group trained by vVO_2max_ (GVO_2_), 2. group trained by V_peak_K_ (GVP). After the adaptation training period, an incremental test was performed at the beginning of each week to adjust training load and to determine the amount of VO_2_ and VCO_2_ fluxes consumed, energy expenditure (EE) and run distance during the incremental test. Mice were submitted to 4 weeks of aerobic exercise training of moderate intensity (velocity referring to 70% of vVO_2max_ and V_peak_K_) in a programmable treadmill. The sessions lasted from 30 to 40 min in the first week, to reach 60 min in the fourth week, in order to provide the mice with a moderate intensity exercise, totaling 20 training sessions.

**Results:** Mice demonstrated increases in VO_2max_ (ml·kg^−1^·min^−1^) (GVO_2_ = 49.1% and GVP = 56.2%), V_peak_K_ (cm·s^−1^) (GVO_2_ = 50.9% and GVP = 22.3%), EE (ml·kg^−0,75^·min^−1^) (GVO_2_ = 39.9% and GVP = 51.5%), and run distance (cm) (GVO_2_ = 43.5% and GVP = 33.4%), after 4 weeks of aerobic training (time effect, *P* < 0.05); there were no differences between the groups.

**Conclusions:** V_peak_K_, as well as vVO_2max_, can be adopted as an alternative test to determine the performance and correct prescription of systemized aerobic protocol training to mice.

## Introduction

The prescription of physical training in mice presents a great challenge owing to the large variety of available testing protocols (Kemi et al., 2002; Ferreira et al., 2007; Ayachi et al., 2016). In particular, running protocols are often used in research involving small rodents because they generally allow the intensity and volume of physical training to be manipulated more easily (Kregel et al., 2007).

Most of the available protocols are adapted from those applied and already tested for prescribed training in humans (Ayachi et al., 2016). Therefore, some variables used to predict human performance, such as maximal oxygen uptake (VO_2max_), velocity associated with its occurrence (vVO_2max_), thresholds related to blood lactate response (lactate threshold, anaerobic threshold, and maximal lactate steady state [MLSS]), and maximum running speed (V_peak_) (Morgan et al., 1989; Bassett and Howley, 2000; Stratton et al., 2009) have also been used in several animals studies with the aim of reproducing the models already developed to evaluate and monitor training performance and prescription in humans (Carvalho et al., 2005; Ferreira et al., 2007; Manoel et al., 2017).

The most classical training prescription method for aerobic training in humans is based on tests using VO_2max_ (ACSM, 2011), which represents the highest rate at which oxygen is extracted in the lungs, transported, and used by the body during maximal exercise (Bassett and Howley, 2000), however, it cannot considered the best predictor of performance (Noakes et al., 1990). In order to more accurately predict endurance performance, vVO_2max_ has emerged and is defined as the minimum velocity at which VO_2max_ is reached in an incremental exercise protocol (Daniels et al., 1984; Morgan et al., 1989; Billat et al., 1994, 1999; Buchheit et al., 2010; Rodrigues et al., 2017).

Although VO_2max_ and vVO_2max_ are consolidated variables to predict performance and to monitor and prescribe aerobic training, their determination requires the use of gas analyzers and a team of researchers familiarized with such instruments, periodic calibration, and high cost. This is true whether the tests are performed in humans or rodents (Fernando et al., 1993; Wisløff et al., 2001). Therefore, reliable and less costly alternatives have been tested and validated for training prescription, such as peak running speed (V_peak_), defined as the maximum velocity reached during an incremental test (Machado et al., 2013). This parameter is strongly correlated with endurance performance in human runners (di Prampero, 1986; Wisløff et al., 2001; Machado et al., 2007; Marcaletti et al., 2011) and presents a good correlation with VO_2max._

However, a comparison of training prescriptions and monitoring according to vVO_2max_ and V_peak_ has not been performed and validated in mice. Such a study may help in reducing the cost and increasing the practicality of training prescription in this animal model. With the above in mind, the aim of this study was to compare the efficiency of an aerobic physical training program prescribed according to either vVO_2max_ or V_peak_K_ in mice. Our hypothesis was that V_peak_K_, as well as vVO_2max_, can be used for training prescription to mice, with the advantage of not requiring expensive equipment.

## Materials and methods

### Animals and experimental design

Twenty Swiss male mice, 60 days old, acquired from the State University of Maringá (UEM), were maintained in individual polypropylene cages, lined with shavings and cleaned weekly, in an automated room for photoperiod control light-dark cycle 12/12-h at 20 ± 24°C. The mice were allowed to feed (Nuvilab Cr1®) and drink water *ad libitum* and food intake and body weight were measured weekly and at the end of the experiment. All procedures were previously approved by the Ethics Committee in Animal Research of the State University of Maringá (UEM) (Maringá, Paraná, Brazil) (protocol n° 033/2014).

Mice were randomly divided into two groups with 10 animals each: a group trained by vVO_2max_ (GVO_2_) and another group trained by V_peak_K_ (GVP). Mice were submitted to familiarization and adaptation to running exercise on a treadmill for 3 days, at an initial velocity of 8 cm·s^−1^ until reaching 16 cm·s^−1^, with an initial duration of 20 min until reaching 30 min.

### Incremental test

The same incremental test was used for the determination of VO_2max_, vVO_2max_, and V_peak_K_, assessed on a motorized treadmill (Panlab®, Barcelona, Spain), adapted from the protocol proposed by Machado et al. (2013) in humans. The warm-up period lasted 5-min at 10 cm·s^−1^, with an initial velocity of 19 cm·s^−1^, followed by an increase of 9 cm·s^−1^, every 3 min until exhaustion, which was characterized by the animal incapacity to keep running in the final third of the streak for more than 10 s (Kregel et al., 2007), 0° slope. The speed unit used was cm·s^−1^ (Kurauti et al., 2016) and the training load was adjusted at the beginning of each training week and the group mean used to determine training intensity.

### Determination of VO_2max_ and VVO_2max_

During the incremental test, gas chamber was collected to determine the VO_2max_ by an air flow control system that allows fine regulation by the LE450 Panlab® gas analyzer (Barcelona, Spain) to determine the amount of VO_2_ and VCO_2_ fluxes consumed, energy expenditure (EE) and run distance during the incremental test; the equipment was calibrated weekly. Before initiating the maximal incremental test, mice remained at rest for 5 min to determine the resting VO_2_ (VO_2rep_) (Billat et al., 2005; Machado et al., 2013). VO_2max_ was expressed in values related to body mass from an allometric adjustment equal to 1 (Taylor et al., 1955, 1981).

Exercise intensity data and VO_2max_ were recorded every second (METABOLISM software, Pan Lab/Harvard Instruments, Spain) and monitored by an external researcher who visually determined the highest VO_2_ reached during the test considered the VO_2max_ (McLaughlin et al., 2010), measured at an average of 5-s intervals. Therefore, vVO_2max_ was determined as the minimal velocity at which the highest VO_2max_ occurred (Billat et al., 1994, 1999).

### Determination of V_peak_K_

V_peak_ was considered the peak running speed obtained during the incremental treadmill test (Machado et al., 2013); if the last stage during the test was not completed, the V_peak_K_ was calculated on the part-time achieved using the equation proposed by Kuipers et al. (2003): V_peak_K_ = (V + t/T x speed increase), where V is the corresponding velocity of the last completed stage (cm·s^−1^), t the time (s) of the uncompleted step and, T the completed step (180 s).

### Training protocol

Mice were submitted to 4 weeks of aerobic exercise training in a programmable treadmill (Inbrasport, Porto Alegre, Brazil) adapted with a support to accommodate 10 mice simultaneously. The training intensity was established at 70% of the maximum speed reached during the incremental test for the determination of the variables, vVO_2max_ and V_peak_K_, for the GVO_2_ and GVP groups, respectively. The 70% maximum speed of training correspond to a moderate exercise session since it presents a high correlation with VO_2max_. (Manoel et al., 2017). Training sessions began at 6 a.m. (lights on), consisting of 1 session per day, 5 times a week (Monday to Friday). The sessions lasted from 30 to 40 min in the first week, from 35 to 50 min in the second week, and 50 to 60 min in the third week, reaching 60 min in the fourth week, in order to submit mice to a moderate intensity exercise, totaling 20 training sessions; the weekly test was considered a training session.

### Data analysis

The sample size was calculated using the G-power software (v.3.1.9.2) (Faul et al., 2007), which demonstrated the necessity of a sample of 11 mice per group for the main variable V_peak_K_; the average effect size was set at 0.25, *P* < 0.05 and 80% power for ANOVA analysis with effect and interaction. All statistical analyses were performed using the Statistica software (v.10, StatSoft Inc., Tulsa, OK, USA). Data are presented as mean ± standard deviation (*SD*). Shapiro-Wilk test was performed to verify data normality. Comparison of the pre-and post- training was made by mixed ANOVA for repeated measurements. To locate the differences, Tukey *post-hoc* was applied if significance was observed. The significance level was set at *P* < 0.05. The effect size was calculated using Cohen equation (*d* = M_1_ –M_2_/*SD*_pooled_) comparing post- vs. pre-training period of vVO_2max_ and V_peak_K_. Values of 0.2, 0.5, and 0.8 indicated a small, medium and large average effect, respectively (Cohen, 1988).

## Results

Table 1 shows significant differences in body weight (g), food intake (g) and food intake corrected by body weight after 4 weeks of training (time effect, *P* < 0.05). Mice showed increased body weight (g) but a reduction of food intake (g) over time in both groups. The food intake after 4 weeks showed a marked reduction of GVP group in comparison to GVO2, although not significant.

**Table 1:** Body weight, food intake and food intake/body weight in pre- and post-training.

	**GVO**_**2**_			**GVP**			***P***		
	**Pre**	**Post**	**Delta (%)**	**Pre**	**Post**	**Delta (%)**	***Time***	***Group***	***Interaction***
Body weight(g)	37.2 ± 2.5	42.5 ± 3.3^*^	14.3 ± 3.7	38.5 ± 2.3	43.6 ± 3.8^*^	12.2 ± 4.7	<**0.05**	0.324	0.514
Food intake (g)	64.9 ± 19.5	55.3 ± 8.3^*^	−10.3 ± 18.3	85.1 ± 35.7	57.9 ± 10.5^*^	−22.2 ± 33.7	<**0.05**	0.121	0.193
Food intake/body weight (g)	17.1 ± 4.4	12.5 ± 1.2^*^	−21.7 ± 17.0	22.1 ± 10.0	13.4 ± 3.1^*^	−30.3 ± 30.9	<**0.05**	0.120	0.257

*Values are presented as mean ± SD. Delta percentage (%) and P-values for time, group and interaction*.

* *P < 0.05 significantly different for time comparison (pre- vs. post-training period)*.

Table 2 demonstrates that 4 weeks of training promoted increases in VO_2max_ (ml·kg^−1^·min^−1^), vVO_2max_ (cm·s^−1^), V_peak_K_ (cm·s^−1^), EE (ml·kg^−1^·min^−1^), run distance (cm), and delta (%) after 4 weeks of aerobic training (time effect, *P* < 0.05). Also, GVO_2_ group presented an interaction effect (*P* < 0.05), indicating the combination of group and time together influenced vVO_2max_. There were no differences between the groups in pre-or post-conditions (group effect).

**Table 2:** Pre- and post-training VO_2max_, V_peak_K_, vVO_2max_, EE and run distance.

	**GVO**_**2**_			**GVP**			***P***		
	**Pre**	**Post**	**Delta (%)**	**Pre**	**Post**	**Delta (%)**	***Time***	***Group***	***Inter-action***
VO_2max_ (ml·kg^−1^·min^−1^)	28.6 ± 3.7	39.9 ± 8.3^*^	42.3 ± 40.4	30.71 ± 3.8	47.9 ± 11.7^*^	49.9 ± 44.5	<**0.05**	0.377	0.616
V_peak_K_ (cm·s^−1^)	29.4 ± 8.2	44.3 ± 10.1^*^	52.3 ± 44.5	31.2 ± 5.6	38.2 ± 7.7^*^	35.5 ± 45.9	<**0.05**	0.281	0.341
vVO_2max_ (cm·s^−1^)	33.4 ± 11.2	51.4 ± 9.67^*^^#^	68.1 ± 53.8	38.8 ± 11.9	43.0 ± 9.0^*^	19.5 ± 50.3	<**0.05**	0.801	<**0.05**
EE (ml·kg^−1^·min^−1^)	200.6 ± 29.2	280.1 ± 56.8^*^	43.4 ± 41.5	211.5 ± 23.7	314.5 ± 86.3^*^	49.9 ± 44.4	<**0.05**	0.347	0.621
Run distance (cm)	13, 954.4 ± 7, 185.1	24, 722.1 ± 10, 026.4^*^	119.0 ± 117.7	12, 859.0 ± 4, 334.7	19, 302.9 ± 6, 709.7^*^	76.8 ± 110.8	<**0.05**	0.197	0.339

*Values are presented as mean ± SD. VO_2max_, maximum oxygen uptake; vVO_2max_, velocity associated with maximum oxygen uptake; V_peak_K_, peak velocity adjust to Kuipers et al. (2003); EE, energy expenditure. Delta percentage (%) and P-values for time, group, and interaction analysis*.

* *P < 0.05 significantly different for time comparison (pre- vs. post-training period)*.

# *P < 0.05 significantly different for interaction comparison (pre- vs. post-training period)*.

**Figure 1:**
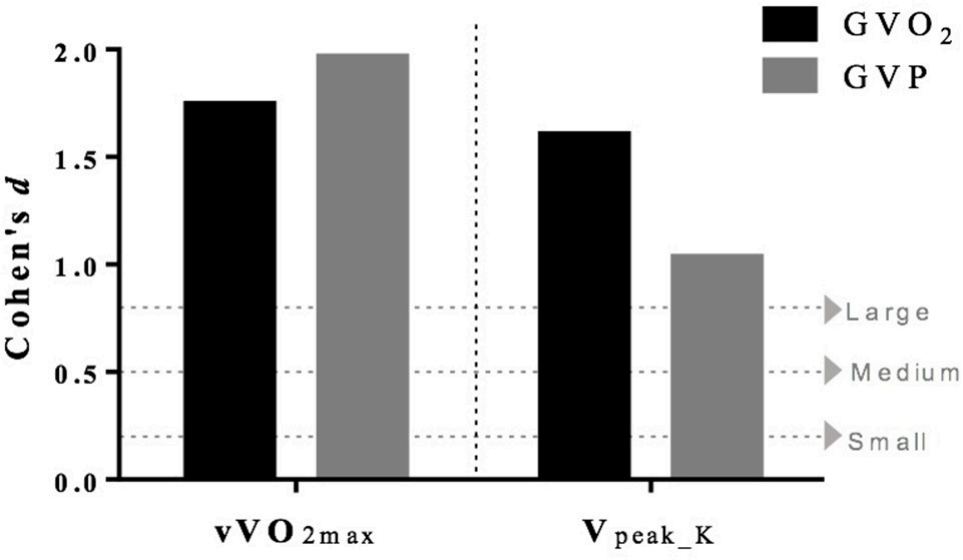
Cohen's effect size comparing post- vs. pre-training period for vVO2max and Vpeak_k.

In order to expand the data analysis beyond the descriptive statistic, we calculated the effect size comparing post- vs. pre-training period for vVO_2max_ and V_peak_K_ to assess the magnitude of findings; Figure 1 shows a large effect in both groups.

## Discussion

The aim of the present study was to compare the efficiency of an aerobic physical training program prescribed according to either vVO_2max_ and V_peak_K_ in mice. Our study demonstrates that both groups, GVO_2_ or GVP, improved VO_2má*x*_, vVO_2max._, and V_peak_K_ after 4 weeks of aerobic training. In addition, V_peak_K_ has been proved a reliable method to prescribe and monitor exercise training. Moreover, training improved performance in both groups and increased EE and the run distance. To our knowledge, this is the first study to address the importance of V_peak_K_ in animal studies.

In the field of exercise physiology, there is great interest in determining the precise intensity of exercise as various research projects use animal models to study the effects of acute and chronic exercise. In this context, several methods to determine training load have been proposed (Kemi et al., 2002; Lerman et al., 2002; Ferreira et al., 2007).

Non-invasive tests report in the literature that use of VO_2max_ as the variable to predict the performance of mice diverge from incremental tests and training protocols. In this sense, establishing a consensus about the results is not possible due to a wide range of reported values (47–94 ml·kg^−1^·min^−1^), according to the slope, magnitude of increase, volume, exhaustion criteria, lineages, age, physical condition, and sex of animals (Dohm et al., 1994; Scott and Houmard, 1994; Lightfoot et al., 2001; Wisløff et al., 2001; Kemi et al., 2002; Ayachi et al., 2016; Kurauti et al., 2016). A few studies have also focused on demonstrating that lineage and sex variations in mice may influence responses in aerobic power (Lightfoot et al., 2010; McLaughlin et al., 2010).

Considering that, it was conceivable that the use of V_peak_K_ would help to minimize the observed differences in VO_2max_ once the variable is more sensitive to changes in physical training in humans (Machado et al., 2013; Manoel et al., 2017). In our study, animals trained according to vVO_2max_ or V_peak_K_ had similar improvements in VO_2max._ and performance over time. In fact, GVO_2_ group presented a higher, although not significant, V_peak_K_. Despite the differences between the two prescription methods it is important to emphasize that the current study demonstrated that animals were able to improve aerobic capacity over time. From a metabolic point of view, other metabolic and structural adaptations may have occurred, hence allowing V_peak_K_-based protocol to be a trustworthy training model for rodents. Therefore, we hypothesize that V_peak_K_ can also contribute to the prescription of exercise in different lineages, sex, and age groups of mice, since it has proved as a training-sensitive variable.

The determination of V_peak_K_, considering the equation proposed by Kuipers et al. (2003), allows evaluating aerobic power and monitoring the effects of training more reliable, as the equation takes into account the last stage completed added to the product of the rate of increase and the fraction of the incomplete last stage, abolishing the influence of subjective judgments or overestimations (Machado et al., 2013). Furthermore, V_peak_K_ is a low-cost, practical, and reliable parameter, which makes it a fundamental tool for future research in the field of exercise physiology, especially considering the known difficulties of assessing VO_2max_ in small rodents (Fernando et al., 1993; Scott and Houmard, 1994; Ayachi et al., 2016).

Although our research was carefully conducted, limitations and shortcomings are unavoidable. The content of muscle glycogen or correlation with blood levels of lactate were not verified during the training period. Also, the training was performed during the day (lights on), opposable to mice habits, which present a predominantly nocturnal behavior.

V_peak_K_ has practical implications for researchers aiming to collect data concerning the effects of training due to its low cost, which is made possible by the fact that it does not require any expensive equipment (i.e., gas analyzer). In summary, V_peak_K_, as well as vVO_2max_, can be adopted as an alternative test to determine the performance and correct prescription of a systemized aerobic protocol training to mice.

## Author contributions

CP: Experiment design, execution of the experiments, writing; PR: Experiment design; GG: Execution of the experiments; DG: Performed statistical analysis; LT: VO2 analysis: SdM: Discussion of data; FM: Conceive the experiments, discussion of data, writing; SP: Concept of the paper, writing.

### Conflict of interest statement

The authors declare that the research was conducted in the absence of any commercial or financial relationships that could be construed as a potential conflict of interest. The reviewer RRC and handling Editor declared their shared affiliation.
